# Diet vs. mental ageing: gender-nutrition approach to higher women’s risk within the PPPM context

**DOI:** 10.1186/1878-5085-5-S1-A145

**Published:** 2014-02-11

**Authors:** Niva Shapira

**Affiliations:** 1 Institute for Nutrition Research, Rabin Medical Center (Beilinson Hospital), Petah Tikva, Israel

## Background

Western ageing, the main predictive measure for developing dementia and Alzheimer’s disease (AD) was found to be associated with increasing of risk factors, i.e. metabolic syndrome/diabetes, oxidative stress, inflammation states, hypertension and homocysteine, and with decreasing endothelial function and production of nitric oxide. Gender is another risk contributing factor for mental decline, with women having higher dementia incidence than in men, suggested to be associated with their higher risk for oxidative damage, i.e. due to inflammation, mitochondrial toxicity, beta-amyloid production, and resulting apoptogenic signals. These patterns especially exemplified in advanced age following menopause, which is known to be a major metabolic turning-stage associated with increasing age-related risks.

## Scientific objectives

The dietary measure that can potentially reduce the risk factors, i.e. reducing glycemic load, n-6:n-3 polyunsaturated fatty acid (PUFA) ratio, and increasing n-9 monounsaturated fatty acids (MUFA), certain vitamins, minerals, antioxidants, fiber, phytosterols and further nutraceuticals.

## Technological approaches

The Mediterranean diet (MeDi) is an eating pattern that has been associated with reduction of western diseases and syndromes, including CVD, metabolic syndrome, diabetes, and cancer, as well as early mental impairment. It has specifically been linked to lowered risk of cognitive decline and AD prevalence and mortality, and further with their predictive factors such as high blood pressure, insulin resistance, and obesity. Although the MeDi combines several foods, micro-, and macro-nutrients already individually proposed as potential protective factors against dementia and predementia syndromes, only recently higher adherence to a Mediterranean-type diet was associated with decreased rate of cognitive decline, of AD and of pre-dementia syndrome and with slowing their progression to overt dementia. Micronutrients supplementations have further suggested protective contribution.

## Results interpretation

The presentation will describe and analyse scientific data supporting predictive measures and risk factors and the nutritional potential for reducing rate of mental decline and explain how adherence to the MeDi may provide nutritional protection. It will further provide data showing women's higher risk for mental decline and present a gender-nutritional prevention approach within the context of PPPM in the health care.

